# From Recognition to Prevention: Modern Approaches to Complication Reduction in Colorectal Surgery

**DOI:** 10.3390/jcm15145412

**Published:** 2026-07-10

**Authors:** Yu-Ting Yeh, Nina Sriram, Waka Yanagisawa

**Affiliations:** 1 Department of General Surgery, Hawkesbury District Hospital, 2 Day Street, Windsor, NSW 2756, Australia; 2 Department of General Surgery, Blacktown & Mount Druitt Hospital, Blacktown Road, Blacktown, NSW 2148, Australia; 3 Department of Surgery, Macquarie University Hospital, 3 Technology Place, Macquarie University, Sydney, NSW 2109, Australia; 4 Department of General Surgery, Westmead Hospital, Western Sydney Local Health District, Sydney, NSW 2145, Australia

**Keywords:** anastomotic leak, colorectal surgery, surgical site infection, colovesical fistula, ERAS, self-expanding metal stent, indocyanine green, laparoscopy, robotic surgery, postoperative complications

## Abstract

Postoperative complications following colorectal surgery—including anastomotic leak (AL), surgical site infection (SSI), perioperative haemorrhage and colovesical fistula—represent major causes of patient morbidity and mortality, prolonged hospitalisation, and healthcare expenditure. This review summarises contemporary evidence across two key domains of complication management—prevention and diagnosis—applied to four major complications (AL, SSI, perioperative haemorrhage, and colovesical fistula), drawn from a comprehensive literature review of recent randomised controlled trials, systematic reviews, meta-analyses, and prospective cohort studies. Preventive strategies discussed include optimisation of surgical techniques (minimally invasive and robotic approaches, indocyanine green perfusion assessment, self-expanding metal stent bridge-to-surgery, and negative pressure wound therapy), modification of patient factors where possible (obesity, anaemia, malnutrition, and immunosuppression), and system-level interventions including Enhanced Recovery After Surgery (ERAS) protocols, perioperative beta-blockade, prehabilitation, and structured quality improvement bundles. Diagnostic strategies have evolved to incorporate biomarker surveillance (CRP and procalcitonin), drain fluid pH analysis, CT imaging (including angiography), endoscopy, and novel digital health tools including wearable monitoring and mobile health applications. Reducing the risk of postoperative complications should involve a multidisciplinary, protocolised approach combining intraoperative technique optimisation with structured perioperative care bundles and close post-discharge surveillance, and centralisation to specialist colorectal surgical units.

## 1. Introduction

Colorectal surgery encompasses a broad spectrum of procedures performed for both benign and malignant disease. Despite significant advances in perioperative care and surgical technique, postoperative complications remain a major source of patient morbidity and mortality, as well as a considerable burden on healthcare systems [1].

Among the most feared complications is anastomotic leak (AL), defined as a defect in the integrity of a surgical join between two hollow viscera, with resultant communication between the intra- and extra-luminal compartments. AL occurs in approximately 3–10% of colorectal resections, with rates varying by anastomotic level, operative urgency, and patient-related risk factors [1,2,3]. The clinical consequences range from small radiologically detected leaks managed conservatively, through to life-threatening faecal peritonitis necessitating emergency reintervention, long-term stoma formation, and impaired oncological outcomes. The economic implications are substantial: AL is associated with significantly prolonged hospital stay, readmission, reoperation, and increased healthcare expenditure [1].

Surgical site infection (SSI) is the most common postoperative complication in colorectal surgery, affecting up to 20% of patients depending on wound classification and patient factors [4]. SSIs encompass superficial incisional, deep incisional, and organ-space infections—the last of which can overlap with an associated AL. Like AL, an SSI increases length of stay, readmission rates, and healthcare expenditure, and is a key quality indicator in colorectal surgery.

Colovesical fistula is an abnormal communication between the colon and bladder. This represents a post-operative complication of both primary colorectal pathology (most commonly diverticular disease or colorectal cancer) and its surgical management. While colovesical fistula is not exclusively a postoperative complication, surgical intervention carries inherent risks of its formation or recurrence.

Perioperative haemorrhage, including both intraoperative and postoperative bleeding, represents a further complication of colorectal surgery. Although less common than AL or SSI, it carries life-threatening consequences and requires specialised preventive and diagnostic strategies. Intraoperative haemorrhage risk is increased in specific contexts such as splenic flexure mobilisation, major mesenteric vessel ligation during D3 lymphadenectomy, and pelvic dissection in proximity to the internal iliac vessels and presacral venous plexus. Careful perioperative management of anticoagulant and antiplatelet therapy, meticulous intraoperative haemostasis, and the selective use of on-table endoscopic evaluation of anastomotic staple-line bleeding are all important preventative strategies with respect to minimising the risk of perioperative haemorrhage. Postoperative haemorrhage is most effectively managed through a stepwise approach, from endoscopic haemostasis and highly selective angiographic embolization, in the suitable cases, through to surgical reintervention with prioritisation of the least invasive effective intervention [5].

The clinical and economic impact associated with these complications is substantial. Anastomotic leak following low anterior resection is associated with significantly prolonged hospital stay, higher rates of 30-day readmission, and increased costs [1]. Surgical site infections similarly prolong hospitalisation, increase readmission rates, and impose a significant burden on the healthcare system. Additionally, colovesical fistula requires complex multidisciplinary management leading to considerable resource utilisation. These findings reinforce the importance of investing in evidence-based preventive strategies, which have the potential to offset significant downstream expenditure and improve patient outcomes.

This review examines the current literature for preventive and diagnostic strategies across four key complications of colorectal surgery (AL, SSI, colovesical fistula, and perioperative haemorrhage) with a particular focus on AL. The literature reviewed includes recent high-quality randomised controlled trials, systematic reviews, and meta-analyses from a comprehensive MEDLINE search. The aim is to provide an update on the latest guidelines and strategies to prevent and diagnose the complications outlined for general and colorectal surgeons and perioperative teams.

## 2. Methodology

We conducted a literature search of Ovid MEDLINE for the past 80 years (end date—5 January 2026). The search included MESH headings and free-text terms across three areas: colorectal surgery, emergency presentations, and postoperative complications. The first domain aimed to capture research related to colorectal surgical procedures, including subject headings for colorectal surgery terms such as “colon,” “rectal,” “rectum,” and “colorect*”. The second domain identified emergency or urgent presentations using headings and free-text terms such as “emergency” and “emergency treatment”. The third domain aimed to capture postoperative complications relevant to this review, including anastomotic leak, surgical site infection, wound infection, haemorrhage and colovesical fistula, combined with terms reflecting prevention, diagnosis, and management.

Paediatric and adolescent populations and case reports were subsequently excluded. Results were limited to English-language publications from the past 10 years and filtered using Ovid’s filters for aetiology, therapy, and diagnosis, returning 189 records. Titles and abstracts were then assessed individually for relevance to anastomotic leak, surgical site infection, colovesical fistula, and perioperative haemorrhage in colorectal surgery. Overall, 109 records were excluded at this stage (one as a duplicate, and the remainder for addressing complications outside the scope of this review). This left 80 records for evaluation, discussion and inclusion in our literature review.

## 3. Preventative Strategies

### 3.1. Colorectal Anastomotic Leak

#### 3.1.1. Surgical Approach: Minimally Invasive and Robotic Surgery

The surgical approach significantly influences anastomotic integrity and postoperative outcomes. Laparoscopic colectomy has been extensively studied as an alternative to open surgery, and evidence supports its role in reducing AL-associated morbidity. A multicentre cohort study by Dauser et al. demonstrated that in the event of AL following right colectomy, patients who had undergone laparoscopic surgery had a shorter time to surgical revision and lower AL-associated mortality compared with those who had open surgery [2]. Laparoscopic surgery has also been associated with shorter hospital stays in colon cancer surgery [6]. A systematic review and meta-analysis of laparoscopic versus open colectomy for obstructing right colon cancer demonstrated lower intraoperative blood loss and shorter length of hospital stay with the laparoscopic approach, although the operative time was longer [7].

Robotic-assisted surgery is a rapidly evolving area in both elective and emergency colorectal surgery. A 2025 systematic review and meta-analysis of 2340 patients by Coco and Leanza found that robotic surgery in acute colorectal emergencies was associated with significantly lower conversion rates to open surgery (OR 0.42) and reduced overall complication rates (OR 0.61) compared with conventional approaches, despite longer operative times [8]. A further systematic review specifically examining robotic versus laparoscopic surgery in complicated diverticulitis found benefits in terms of conversion rates and short-term outcomes, though the evidence base is still limited [9]. While robotic surgery is promising, further high-quality randomised controlled trials (RCTs) are required to define its role in emergency and complex colorectal surgery.

#### 3.1.2. Primary Anastomosis Versus Hartmann’s Procedure

The choice between primary anastomosis (with or without proximal diversion) and Hartmann’s procedure (HP) in emergency colorectal surgery has been a subject of ongoing debate. HP has historically been favoured in the emergency setting due to concerns about anastomotic integrity and higher risks of AL in the presence of contamination or haemodynamic compromise.

A retrospective cohort study by de Almeida Leite et al., using the ACS-NSQIP database and including 25,458 patients with acute diverticulitis, found that primary anastomosis was associated with lower rates of medical-related morbidity and mortality compared with HP, even in patients with septic shock [10]. Notably, primary anastomosis without a diverting ileostomy was associated with fewer unplanned readmissions than the ileostomy group in the septic shock cohort. A comparative study by Aras further supports primary resection and anastomosis over HP in obstructing colorectal cancer, demonstrating shorter hospital stays, lower complication rates, and comparable overall survival [11]. Collectively, these data support a selective approach to HP, reserving it for the most physiologically compromised patients, with primary anastomosis representing an increasingly viable and preferred option in appropriately selected patients.

#### 3.1.3. Intraoperative Colonic Lavage

In left-sided colonic emergencies, the choice between intraoperative colonic lavage (IOCL) and manual decompression (MD) to prepare a distended, unprepared bowel for primary anastomosis still remains a topic of debate amongst surgeons. A retrospective study from Pisa University Hospital evaluating 70 patients found no significant difference in anastomotic leakage, SSI, overall morbidity, or length of hospital stay between IOCL and MD, although IOCL was associated with shorter ICU stay and longer operative time [12]. These findings suggest that MD remains a reasonable and simpler alternative in the majority of emergency cases. Importantly, evidence suggests that IOCL in isolation does not significantly reduce the rate of anastomotic leak; its benefit in this regard appears to be dependent on concurrent protective measures, such as side-to-end anastomosis and/or the addition of a diverting stoma, which together may mitigate the consequences (but not the incidence) of anastomosis leak. Similarly, a review of seven trials (449 patients) comparing IOCL with MD alone in obstructed left-sided colorectal cancer found no significant difference in anastomotic leak rates or mortality between the two techniques [13]. This supports manual decompression as a comparable and less technically demanding alternative when primary anastomosis is planned in the emergency setting.

#### 3.1.4. Drain Placement

The routine placement of intraperitoneal drains following colorectal surgery is widely practiced, yet evidence for their efficacy in detecting or alleviating the clinical impact of AL remains uncertain. The international prospective COMPASS cohort study evaluated drain use in emergency colorectal surgery and found no significant association between drain placement and reduced complications or improved outcomes [14]. The blanket use of perioperative drains cannot therefore be recommended, and their use should be guided by individual operative findings and surgeon judgement rather than applied as a universal prophylactic measure.

#### 3.1.5. Self-Expanding Metal Stents as a Bridge to Surgery

Self-expanding metal stents (SEMS) have been proposed as an alternative to emergency surgery in malignant large bowel obstruction (MLBO), acting as a bridge to elective resection. By converting an emergency procedure into an elective resection, the aim is to allow decompression of the bowel, patient optimisation, and staging—thereby facilitating primary anastomosis and avoiding stoma formation.

A 2025 retrospective cohort study by Farrow et al. demonstrated the safety and efficacy of a stent-first approach for both left- and right-sided MLBO, with acceptable technical success rates and complication profiles [15]. Multiple systematic reviews and meta-analyses of RCTs comparing SEMS bridge-to-surgery with emergency resection have consistently shown higher rates of primary anastomosis and lower stoma rates with SEMS, with comparable or reduced short-term morbidity [16,17,18,19]. A network meta-analysis comparing four treatment strategies for acute left malignant colonic obstruction—including SEMS, decompressing stoma, emergency resection, and staged resection—found SEMS associated with superior short-term outcomes and similar 5-year overall and disease-free survival [20].

The optimal timing of surgery following SEMS insertion is an additional consideration. A 2025 multicentre study concluded that a minimum interval of two to four weeks between stenting and resection was associated with lower complication rates and higher rates of successful primary anastomosis [21]. For palliation in unresectable disease, SEMS offers effective symptom control with reasonable long-term outcomes [22,23]. However, concerns persist regarding oncological safety, including the risk of stent-related perforation, tumour seeding, and potential compromise of pathological staging. A retrospective cohort study found higher complication rates with SEMS, including perforation and stent migration, particularly with longer stent indwelling times [24]. The ESCO multicentre RCT similarly reported a higher rate of 30-day complications in the SEMS arm compared with emergency surgery, highlighting the importance of careful patient selection [25].

A comparison of decompressing stoma versus SEMS as a bridge-to-surgery found comparable surgical outcomes, but decompressing stoma was associated with higher primary anastomosis rates in some analyses [26]. A prospective multicentre Japanese study of 312 patients found SEMS technically feasible and safe as a bridge-to-surgery, with a technical success rate of 91.3% and clinical success of 88.5% [27]. Similarly, a retrospective evaluation confirmed shorter postoperative stays and lower stoma rates with SEMS compared with emergency surgery [28].

The ESCO multicentre RCT demonstrated that colonic stenting reduced stoma rates compared with emergency surgery in MLBO, though at the cost of a higher rate of 30-day complications in the SEMS group [25]. Taken together, SEMS is a valuable tool in appropriately selected patients, particularly where the clinical scenario permits delay and the patient is physiologically stable.

#### 3.1.6. Indocyanine Green Fluorescence Angiography

Vascular supply is a key determinant of anastomotic healing. A prospective study by Norooz et al. examined the association between calcium scoring of the descending aorta and major pelvic vessels—a validated measure of atherosclerotic burden—and the incidence of AL following colorectal anastomosis. The study found a significant positive correlation between calcium score and AL rate, suggesting that preoperative assessment of vascular disease burden may offer a novel predictive tool for identifying high-risk patients in whom closer monitoring or protective measures are warranted [29].

Adequate perfusion at the anastomotic site is a prerequisite for healing, and ischaemia is a recognised risk factor for AL. Indocyanine green (ICG) near-infrared fluorescence angiography (NIRF) permits real-time, intraoperative assessment of intestinal perfusion prior to and after anastomotic formation. A prospective study by Impellizzeri et al. demonstrated a statistically significant reduction in AL rate in patients who underwent NIRF/ICG-guided perfusion assessment (2.5% vs. 9.6% in the control group), leading to intraoperative change at the level of anastomosis in a proportion of cases [30]. ICG fluorescence angiography represents a promising adjunct in reducing AL, particularly in cases where bowel vascularity may be compromised.

#### 3.1.7. Optimisation of Patient Risk Factors

Modifiable patient-related risk factors for AL and other postoperative complications are well established. A systematic review and meta-analysis of preoperative risk factors for AL following colectomy for colorectal cancer identified obesity, male sex, smoking, diabetes, ASA score, preoperative bowel obstruction, and tumour characteristics as significant predictors of AL [31]. Optimisation of these factors prior to elective surgery is a cornerstone of preoperative assessment.

Obesity is a particularly important modifiable risk factor. Multiple studies have demonstrated that elevated BMI is independently associated with increased rates of AL, SSI, and other postoperative complications [32,33,34]. A French multicentre cohort study confirmed that obese and morbidly obese patients undergoing emergency surgery for obstructive colon cancer had significantly higher morbidity rates compared with normal-weight counterparts [34]. Weight optimisation prior to elective surgery, ideally as part of a multidisciplinary approach, should therefore be encouraged where clinically feasible.

Inflammatory bowel disease, and particularly Crohn’s disease, confers a significantly elevated risk of postoperative complications. An ACS-NSQIP database analysis found that immunosuppressed patients with Crohn’s disease had significantly higher rates of major complications following colonic resection, including anastomotic complications and wound infections, compared with immunocompetent individuals with Crohn’s [35]. Careful perioperative planning, including consideration of the timing of surgery relative to immunosuppressive therapy and nutritional optimisation, is essential in this population. Preoperative nutritional status is similarly important; low albumin levels have been identified as an independent predictor of complications including AL and SSI in several analyses [36].

Preoperative anaemia is common in colorectal cancer patients and is independently associated with increased transfusion requirements and postoperative complications. A retrospective cohort study by Kangaspunta et al. demonstrated that preoperative intravenous iron infusion in anaemic patients undergoing colon resection significantly reduced postoperative complications and postoperative anaemia, without increasing the risk of adverse events [37]. Preoperative intravenous iron infusion should therefore be considered part of the routine optimisation pathway for anaemic surgical patients where time permits.

The presence of atrial fibrillation (AF) is associated with worse outcomes following colorectal surgery, including increased morbidity and mortality [38]. Perioperative optimisation of cardiac rhythm and rate should be part of a comprehensive preoperative assessment in patients with known AF.

#### 3.1.8. Perioperative Beta-Blockade

Adrenergic hyperactivity is implicated in postoperative immune dysregulation and may increase the risk of infective complications. Two studies by Ahl et al. examined the role of preoperative beta-blocker therapy in emergency colon cancer surgery. The first demonstrated that regular preoperative beta-blockade was associated with a significant reduction in 30-day mortality after emergency colonic cancer surgery [39]. A subsequent study found that beta-blocker therapy was associated with a reduction in severe postoperative complications and an improvement in long-term survival following emergency surgery for colon cancer, an effect that was independent of cardiovascular comorbidity [40]. These findings suggest a potential role for perioperative beta-blockade as a preventive strategy in high-risk colorectal surgical patients; however, further robust evidence is needed to support this.

#### 3.1.9. Enhanced Recovery After Surgery (Eras)

Enhanced Recovery After Surgery (ERAS) protocols represent a multimodal, evidence-based approach to perioperative care that aims to minimise the effects of surgical stress, maintain physiological function, and accelerate postoperative recovery. ERAS was initially developed for elective colorectal surgery and is now standard of care in that setting. Its application in emergency colorectal surgery has attracted increasing interest.

A meta-analysis by Ahmed et al. comparing ERAS with conventional care in emergency colorectal surgery found that ERAS was associated with significant reductions in length of hospital stay, overall complication rates, and postoperative ileus, without increasing readmission rates [41]. A systematic review and meta-analysis focused on emergency resection for obstructive colorectal cancer also demonstrated reduced morbidity and shorter hospital stay with ERAS protocols [42]. A single-centre retrospective cohort study further confirmed that ERAS principles could be safely applied to emergency patients, with improved outcomes compared with conventional management [43]. Critically, early mobilisation is a key component of ERAS; a retrospective cohort study confirmed the feasibility of early postoperative mobilisation within an established ERAS programme and its association with shorter hospital stay [44].

#### 3.1.10. Prehabilitation and Obstruction Management Protocols

Patients presenting with colorectal obstruction often have suboptimal nutritional and physical states, increasing the risks of emergency surgery. Prehabilitation, the process of optimising functional capacity and nutrition prior to planned surgery, has the potential to reduce postoperative morbidity. Two related studies by Fahim et al. described a multimodal protocol for bowel obstruction patients, incorporating SEMS for decompression followed by prehabilitation and staged elective resection. A pilot study showed promise, and a subsequent multicentre prospective study confirmed significantly reduced emergency surgery rates (from 65% to 15%), lower 30-day morbidity, and lower mortality in patients managed according to this protocol compared with historical controls [45,46]. These findings support a protocol-driven approach to bowel obstruction that incorporates bridge-to-surgery and prehabilitation as complementary strategies.

#### 3.1.11. Diverting Stoma as Anastomotic Protection

A diverting ileostomy or colostomy is frequently formed to protect a low colorectal or coloanal anastomosis from the consequences of AL. However, ileostomy itself carries significant morbidity—including dehydration, renal impairment, peristomal complications, and the need for a subsequent reversal procedure. A randomised controlled trial by Kim et al. evaluated a faecal diversion device (FDD)—a transanal, rectal tube-based system—as an alternative to diverting ileostomy following rectal anastomosis, demonstrating comparable safety and effectiveness with a significantly lower complication profile and avoidance of the morbidity associated with stoma formation and reversal [47].

Where diversion is deemed necessary, loop colostomy can be considered as an alternative to loop ileostomy in select patients with left-sided obstruction. A retrospective cohort study by Amelung et al. demonstrated that loop colostomy as a bridge to surgery for acute left-sided colonic obstruction was associated with acceptable morbidity and offered a valuable decompression strategy prior to elective resection [48].

#### 3.1.12. Reducing Anastomotic Leak in the Emergency Setting

In the emergency colorectal surgical setting, the inclusion of perforated diverticulitis and acute large bowel obstruction imposes specific risks for both anastomotic leak and perioperative haemorrhage that are not fully addressed by strategies developed in the elective context.

In patients presenting with perforated diverticulitis, large bowel obstruction and faecal peritonitis, the combination of bowel contamination, haemodynamic compromise, and physiological derangement renders primary anastomosis technically and biologically challenging. Nevertheless, as described above, evidence supports primary anastomosis with selective proximal diversion as a viable and increasingly preferred alternative to Hartmann’s procedure in appropriately selected patients, even in the context of septic shock, with lower medical morbidity and comparable mortality [10]. The decision to proceed with anastomosis in this setting must incorporate careful intraoperative assessment of bowel perfusion—a domain in which ICG fluorescence angiography offers particular value, having been shown to reduce AL rates by enabling real-time identification of ischaemic anastomotic tissue prior to closure [30]. Furthermore, risk factors including obesity, hypoalbuminemia, immunosuppression, tumour burden and preoperative anaemia independently amplified anastomotic leak risk in this context, which should affect the intraoperative decision-making process [31,35,36,37]. When primary anastomosis is performed in a contaminated field, as seen in an emergency setting, the addition of a diverting stoma and omental exclusion are important safety nets, mitigating the clinical consequences of AL (i.e., recurrent faecal peritonitis) without necessarily preventing its occurrence completely [47].

In patients with acute large bowel obstruction unsuitable for immediate resection, SEMS bridge-to-surgery enables physiological optimisation and nutritional recovery prior to elective anastomosis—strategies shown to reduce anastomotic failure and stoma rates compared with emergency resection [15,16,20,45,46].

### 3.2. Wound Infections (Surgical Site Infection)

#### 3.2.1. Surgical Approach and Technique

As with AL, the surgical approach influences SSI rates significantly. A systematic review and meta-analysis of laparoscopic versus open surgery in patients with obstructive colorectal cancer following stent placement demonstrated lower SSI rates with laparoscopic colectomy compared with open surgery, in addition to reduced blood loss and shorter hospital stays [49]. Open surgery is associated with higher rates of SSI due to larger wound exposure, greater tissue trauma, and prolonged operative time.

Surgical technique adjuncts have also been studied in the context of SSI prevention. The use of a circumferential wound retractor (Alexis retractor) in emergency colorectal surgery was associated with a significant reduction in SSI rates in a retrospective study, likely by reducing wound contamination with bowel contents during mobilisation and anastomosis [50]. Skin antisepsis practice is also relevant: a prospective cohort study found no additional benefit from three sequential antiseptic paints over two in terms of SSI prevention, suggesting that two-step preparation may be sufficient [51].

Primary anastomosis with resection confers a lower risk of organ space or anastomotic SSI compared with non-restorative procedures in some analyses; a systematic review and meta-analysis found that resection with primary anastomosis for perforated diverticulitis with peritonitis was associated with comparable or lower rates of organ-space SSI compared with HP, with lower stoma rates and shorter hospital stays [52].

#### 3.2.2. Negative Pressure Wound Therapy

Negative pressure wound therapy (NPWT), involving the application of controlled subatmospheric pressure to a wound, has been evaluated as a preventive strategy for SSI following colorectal surgery, with particular interest in use for high-risk and grossly contaminated wounds. A 2025 systematic review and meta-analysis of controlled trials by Wang et al. found that NPWT significantly reduced SSI rates following colorectal surgery compared with standard wound care [53]. A systematic review by Bastawisy et al. similarly concluded that NPWT was effective in reducing SSI in abdominal surgery [54].

The specific application of NPWT to closed laparotomy incisions was examined in a JAMA Surgery systematic review and meta-analysis by Sahebally et al., which found a significant reduction in SSI rates with prophylactic NPWT compared with standard dressings in general and colorectal surgery [55]. A prospective multicentre RCT (SWIPE IT) evaluating NPWT versus standard closure on closed abdominal incisions in colorectal surgery found reduced dehiscence rates with NPWT, though without a statistically significant reduction in SSI rates [56]. A further retrospective cohort study evaluated the PICO 7 NPWT device in high-risk patients undergoing colorectal resections and emergency laparotomy, finding higher SSI rates in the control group despite appropriate selection, suggesting a benefit particularly in high-risk wounds [57].

In the context of delayed primary closure following emergency laparotomy for colorectal perforation, a multicentre retrospective cohort study found that NPWT reduced incisional SSI rates compared with conventional delayed closure [58]. An RCT in grossly contaminated emergency abdominal surgeries confirmed that NPWT-assisted delayed primary closure significantly reduced SSI compared with conventional delayed closure [59].

#### 3.2.3. Mechanical Bowel Preparation

The use of preoperative mechanical bowel preparation (MBP) to reduce SSI following colon surgery remains contentious. The landmark MOBILE multicentre RCT demonstrated that mechanical and oral antibiotic bowel preparation (MOABP) was superior to no bowel preparation in reducing SSI rates in elective colectomy [60]. However, questions remain about the optimal components of bowel preparation and whether MBP alone (without oral antibiotics) confers sufficient benefit. An ongoing Canadian multicentre RCT (the Ghuman et al. protocol) is designed to provide higher-quality evidence on this question, and its results are awaited [61].

#### 3.2.4. Intraoperative Measures

Several intraoperative techniques have been studied for SSI prevention. Maintaining normothermia throughout surgery has been recommended in current guidelines, and a cohort study by Fahim et al. confirmed that intraoperative hypothermia was associated with significantly higher rates of SSI and other postoperative complications in colorectal cancer surgery [62]. Active patient warning measures are therefore an important component of SSI prevention.

Perioperative hyperoxygenation—the administration of an 80% fraction of inspired oxygen (FiO_2_) compared with the standard 30–35%—has been evaluated in an RCT of emergency abdominal surgery by Yerra et al. Hyperoxygenation significantly reduced SSI rates compared with standard oxygenation (9.1% vs. 28.6%), supporting its use as a simple and cost-effective preventive measure [63].

Insufflation of the open surgical wound with warm humidified CO_2_ (HumiGard system) was evaluated in an Australian RCT by Arachchi et al. in open colorectal surgery. While conceptually promising as a means of reducing wound desiccation and bacterial contamination, the trial did not demonstrate a statistically significant reduction in SSI rates, and this technique cannot therefore be routinely recommended based on current evidence [64].

#### 3.2.5. Weight Optimisation and Medical Comorbidities

As with AL, obesity is a well-established independent risk factor for SSI following colorectal surgery. Multiple analyses confirm the association between elevated BMI and increased rates of superficial and deep SSI [32,33,34,65]. Preoperative weight loss should be encouraged where feasible, particularly prior to elective procedures.

Medical comorbidities including Crohn’s disease, immunosuppression, diabetes, and hypoalbuminaemia are important modifiable and non-modifiable risk factors for SSI. Crohn’s patients on immunosuppression have significantly elevated SSI risk [35], and nutritional optimisation (including correction of hypoalbuminaemia) should be a preoperative priority [36].

#### 3.2.6. Iron Infusion and Perioperative Anaemia Management

Anaemia and iron deficiency impair wound healing and immune function, increasing SSI susceptibility. Preoperative intravenous iron infusion in anaemic patients undergoing colorectal surgery has been associated with reduced postoperative complications, as described above [37], and represents a simple and potentially impactful intervention in the preoperative optimisation pathway.

#### 3.2.7. Emergency Surgery

Emergency surgery is an independent risk factor for SSI following colorectal procedures. An NSQIP database analysis confirmed significantly higher SSI rates in emergency versus elective partial colectomy [4], reflecting the contribution of bowel contamination, wound classification, and patient physiological status. Early emergency colectomy for steroid-refractory acute severe ulcerative colitis (ASUC) also reduces the burden of SSI compared with delayed surgical intervention, as prolonged medical management is associated with greater physiological deterioration and higher complication rates [66,67].

### 3.3. Colovesical Fistula

Colovesical fistula is most commonly a consequence of complicated diverticular disease, colorectal cancer, Crohn’s disease, or previous pelvic irradiation, and occurs in approximately 5–10% of patients with complicated diverticulitis. The prevention of fistula in the context of colorectal surgery is primarily achieved through strategies that reduce the incidence of its underlying causes and that minimise the likelihood of inadvertent fistula formation during operative repair.

Minimally invasive surgery may facilitate precise dissection in the pelvis and reduce the risk of inadvertent injury to the bladder during mobilisation [8]. Patient-level risk factors for fistula, such as obesity, Crohn’s disease, prior pelvic surgery, and immunosuppression, should be optimised preoperatively [31,32,33,34,35]. Early colectomy for fulminant ulcerative colitis reduces the effect of physiological and inflammatory factors that predisposes to fistula formation in that population [66]. Surgeon specialisation improves outcomes in the management of fistulating disease, and preoperative iron optimisation should be considered in anaemic patients [37].

### 3.4. Elective and Emergency Perioperative Haemorrhage

Intraoperative and postoperative haemorrhage represents an important and potentially life-threatening complication of colorectal surgery. Preventive strategies begin in the preoperative period with careful assessment and management of anticoagulant and antiplatelet therapy. Bridging strategies and timing of cessation should be individualised according to the indication for anticoagulation, the thrombotic risk profile, and the urgency of the surgical procedure, in accordance with current haematological and cardiology guidelines.

Intraoperatively, haemorrhage risk is heightened in specific procedural contexts. Splenic mobilisation when performed as part of extended left hemicolectomy, total colectomy, or mobilisation of the splenic flexure carries a recognised risk of capsular tear and splenic haemorrhage, requiring careful retraction technique and a low threshold for splenectomy where haemostasis cannot be achieved. Major mesenteric dissection, including D3 lymphadenectomy for right-sided colon cancer, involves the ligation of major mesenteric vessels, and meticulous dissection with secure vascular control is essential to prevent intraoperative haemorrhage. Similarly, pelvic lymph node dissection in rectal cancer surgery involves dissection in proximity to the internal iliac vessels and presacral venous plexus, where bleeding can be difficult to control and may be significant.

Intraoperative endoscopic evaluation of anastomotic mucosal bleeding, performed via on-table colonoscopy or flexible sigmoidoscopy following anastomotic formation, allows direct visualisation and endoscopic treatment of intraluminal anastomotic haemorrhage before closure, reducing the risk of clinically significant postoperative bleeding. This technique is relevant following low anterior resection, where anastomosis may be at risk of mucosal ischaemia or staple-line bleeding. Should postoperative haemorrhage occur, a stepwise management approach, incorporating early endoscopic evaluation and haemostasis, selective angiographic embolisation, and reoperation for refractory cases, is consistent with the latest evidence and the principle of preferentially utilising the least invasive effective intervention [5].

The emergency context is associated with increased bleeding risk due to distended, friable bowel, attenuated tissue planes secondary to inflammation, and suboptimal vascular control in the setting of contamination or prior surgical intervention. Active intra-oeprative physiology optimisation—warming the patients, correcting metabolic acidosis and administrating tranexamic acid—reduces the coagulopathy and limits haemorrhage. Surgical hemostatic products are vital adjuvants to achieve extraluminal haemorrhage.

Intraoperative on-table endoscopic evaluation of the anastomotic staple line following colorectal anastomosis enables direct visualisation and identification of intraluminal bleeding prior to abdominal closure, reducing the risk of clinically significant postoperative endoluminal haemorrhage [5]. Should postoperative endoluminal bleeding arise, a stepwise approach—prioritising endoscopic haemostasis before reoperation—can be undertaken. Selective angiographic embolisation before reoperation plays an additional important role in controlling extraluminal bleeding.

The emergency colorectal setting demands a protocolised, individualised, and technically rigorous approach to reduce both anastomotic leak and perioperative haemorrhage, integrating patient selection, intraoperative perfusion assessment, and structured postoperative surveillance within an ERAS framework [41,42,43].

## 4. Diagnostic Strategies

### 4.1. Colorectal Anastomotic Leak

#### 4.1.1. Clinical Recognition and the Challenge of Early Diagnosis

Anastomotic leak may present on a spectrum from subtle clinical signs such as prolonged ileus, tachycardia, and low-grade fever through to fulminant peritonitis and septic shock. The early clinical signs of AL are often non-specific and may overlap with other postoperative complications, making early and accurate diagnosis challenging. Clinical suspicion should be high in any patient who fails to progress as expected postoperatively or who demonstrates unexplained deterioration.

Delayed diagnosis of AL is associated with poorer clinical outcomes, including higher rates of surgical intervention, prolonged ICU stay, and mortality. As a result, developing reliable, early diagnostic tools remains a priority. The CONDOR (COmplication detectioN after cOloRrectal surgery) study, a prospective multicentre clinical diagnostic study, was designed to create a structured decision algorithm for the early detection of AL following elective colorectal surgery, incorporating both biochemical and clinical parameters [68]. Tools of this type represent an important step toward standardised, protocol-driven care.

#### 4.1.2. Biomarkers: Crp and Procalcitonin

C-reactive protein (CRP) and procalcitonin (PCT) are acute-phase inflammatory markers that increase in the context of infection and inflammation. Their role as early biomarkers of AL following colorectal surgery has been the subject of significant research.

A systematic review and meta-analysis of studies examining CRP as a predictor of AL by Yeung et al. found that elevated CRP on postoperative days 3–5 was significantly associated with AL, with day 3 and day 4 CRP demonstrating the highest predictive value. A CRP threshold of approximately 150 mg/L on postoperative days 3–4 was identified as clinically useful for risk stratification [69].

A prospective observational study by Baeza-Murcia et al. evaluated both CRP and PCT as early diagnostic tools for AL in patients undergoing elective colorectal surgery. Both markers were significantly elevated on postoperative days 3 and 5 in patients who subsequently developed AL. The combination of CRP and PCT was proposed as a complementary diagnostic strategy, with PCT marking earlier physiological changes and CRP providing sustained elevation as a marker of established sepsis [70]. Routine measurement of these biomarkers in the early postoperative period should be considered as part of a structured AL surveillance protocol.

#### 4.1.3. Drain Fluid Ph

The biochemical analysis of perioperative drain fluid represents a minimally invasive diagnostic adjunct for AL detection. A prospective analysis by Molinari et al. of 173 patients undergoing colorectal surgery demonstrated that drain fluid pH on postoperative day 1 (POD1) was significantly lower in patients who subsequently developed AL compared with those who did not. A drain fluid pH below a defined threshold on POD1 was shown to be a useful negative predictive tool—a normal pH effectively excluding the diagnosis of clinically significant AL [71]. This simple, bedside investigation represents a valuable and low-cost complement to clinical assessment and biomarker surveillance.

#### 4.1.4. CT Imaging

CT of the abdomen and pelvis with rectal contrast is the imaging modality of choice in the investigation of suspected AL. CT findings associated with postoperative complications in acute obstructive colonic cancer surgery (including extraluminal gas, perianastomotic fluid collections, and bowel wall discontinuity) were systematically evaluated by Pezzullo et al., who identified specific CT prognostic signs predictive of poor outcomes [72]. Contrast-enhanced CT permits characterisation of the extent of the leak, identification of associated collections, and assessment of sepsis source—all of which guide subsequent management decisions.

#### 4.1.5. Fluoroscopic Water-Soluble Contrast Enema

Water-soluble contrast enema (WSCE) can be used as a complementary diagnostic tool, particularly for the assessment of anastomotic integrity prior to ileostomy reversal and in cases where CT findings are equivocal. WSCE provides direct visualisation of the anastomosis and can identify subtle defects not apparent on cross-sectional imaging.

#### 4.1.6. Remote Monitoring, Wearables, and Digital Health

As hospital stays following colorectal surgery shorten under ERAS protocols, postoperative complications, including AL, increasingly manifest following discharge. The development of digital health tools to capture clinical deterioration in the community is therefore of growing importance.

A prospective study by Leenen et al. evaluated remote monitoring of continuous vital sign measurements using wearable devices (including heart rate, respiratory rate, and temperature) in patients discharged following colorectal surgery. The study demonstrated that wearables were feasible in this patient group and identified episodes of physiological deterioration that preceded emergency department presentations and readmission [73]. These data suggest that wearable technology has the potential to serve as an early warning system for AL and other complications in the post-discharge period.

Digital health applications as tools for complication detection are also under investigation. The MobiMD trial, a randomised controlled trial of a self-monitoring mobile application, was designed to evaluate whether structured digital patient monitoring could reduce hospital readmissions for complex surgical patients—including those undergoing colorectal surgery [74]. A study by Sigvardt et al. examining the frequency of emergency medical service contacts following surgical hospital admissions found a significant proportion of post-discharge deterioration events, highlighting the opportunity for remote monitoring interventions [75]. Home care visits by community nurses following emergency colorectal surgery have similarly been evaluated as a means of reducing loss to follow-up and enabling earlier identification of postoperative complications, demonstrating significantly improved follow-up rates compared with standard care [76].

### 4.2. Wound Infections

#### Clinical Assessment and Surveillance Tools

SSI surveillance following colorectal surgery is an important quality indicator. Traditional surveillance relies on clinical wound review, but this approach has limitations in the context of shorter hospital stays and day-case procedures. Shitrit et al. developed and validated a semi-automated surveillance screening tool for SSI following colorectal, orthopaedic, and gynaecological surgery, demonstrating good sensitivity and specificity for SSI identification when applied to electronic health record data [77]. Automated surveillance tools of this type may enable more systematic post-discharge SSI monitoring.

Remote monitoring strategies applicable to AL detection, including wearables, mobile health applications, and home care visits, are equally relevant to SSI surveillance [73,74,75,76]. Early identification of wound changes following discharge enables timely community management and may prevent escalation to more serious complications.

CT imaging remains the gold standard for the evaluation of deep incisional and organ-space SSI, with the ability to characterise collections, assess fascial integrity, and guide percutaneous drainage [72].

### 4.3. Colovesical Fistula

#### Diagnostic Evaluation

The clinical presentation of colovesical fistula typically includes pneumaturia, faecaluria, and recurrent urinary tract infections. CT of the abdomen and pelvis with oral and intravenous contrast is the first-line imaging investigation, identifying communication between the bladder and colon, associated inflammatory change, and underlying aetiology [72]. CT cystography may further delineate the fistula tract.

Cystoscopy provides direct visualisation of the bladder mucosa, identifying the fistula orifice and permitting biopsy to determine aetiology (i.e., malignant vs. benign). Flexible sigmoidoscopy or colonoscopy complements cystoscopy in assessing the colonic side of the fistula, particularly where malignancy must be excluded.

Remote monitoring strategies relevant to general postoperative complications, including wearables and digital health tools, may also capture the systemic inflammatory response associated with unrecognised or developing fistulas, though colovesical fistula is more commonly a subacute or chronic presentation than an acute surgical emergency [73,74,75].

### 4.4. Perioperative Haemorrhage

The diagnosis of perioperative haemorrhage relies on a combination of clinical assessment, haemodynamic monitoring, and targeted investigations. Intraoperatively, active bleeding is identified directly under visualisation, and the surgeon should maintain a systematic approach to vascular control and haemostasis throughout the procedure. Postoperative haemorrhage may present with haemodynamic instability, falling haemoglobin serial full blood counts, increasing drain output of frank blood, or per-rectal bleeding. A low threshold for investigation is essential in any patient with unexplained physiological deterioration in the early postoperative period. CT angiography of the abdomen and pelvis is the investigation of choice for suspected postoperative haemorrhage where the patient is haemodynamically non-compromised, enabling precise localisation of the bleeding source and guiding subsequent endoscopic, radiology-guided or surgical intervention [72]. In cases of per-rectal or anastomotic haemorrhage, colonoscopy or flexible sigmoidoscopy provides both diagnostic and therapeutic capability, allowing direct visualisation of the bleeding point and endoscopic haemostasis. When endoscopic access is limited or haemostasis cannot be achieved, highly selective mesenteric angiography with embolisation offers a minimally invasive alternative prior to surgical reoperation. However, the risk of anastomotic leak may increase [5]. Serial haemoglobin monitoring, vital sign monitoring, and close clinical review in the early postoperative period remain the foundation of early haemorrhage recognition, and wearable monitoring technology may offer additional utility in the post-discharge setting [73,75].

## 5. Discussion

This review summarises contemporary evidence across the prevention and diagnosis of AL, SSI and colovesical fistula in colorectal surgery. Multiple areas for discussion are highlighted from the results of the review of the recent literature.

First, prevention is significantly more effective (clinically and economically) than treatment. The significant reductions in AL rates achievable through system-level practice change (as demonstrated by Iversen et al. [78]) and ERAS protocols [41,42] emphasise the importance of structured perioperative care. Individual preventive measures, including ICG fluorescence angiography [30], perioperative beta-blockade [39,40], prehabilitation [45,46], and preoperative risk optimisation [31,32,35,37], each contribute incrementally to complication reduction.

Second, patient selection and individualised risk stratification are central to surgical decision-making. The choices between primary anastomosis and Hartmann’s procedure [10,11], between SEMS bridge-to-surgery and emergency resection [15,16,17,20], and between diverting stoma and no diversion [47] all depend critically on accurate risk assessment. Tools such as calcium scoring [29], preoperative biomarker assessment, and nomogram-based prediction models [79] may enhance this process.

Third, the landscape for the use of specialised diagnostic tools is a rapidly changing space in colorectal surgery. Biomarker-based surveillance using CRP and PCT [69,70], drain fluid pH analysis [71], and the development of early detection algorithms [68] provide clinicians with objective tools to further support clinical judgement. The emergence of wearable technology [73] and mobile health applications [74] as a means of capturing post-discharge clinical deterioration represents a shift in postoperative surveillance, relevant to a healthcare environment in which early discharge is becoming increasingly common practice.

Finally, surgeon and system-level factors are critical determinants of outcomes. The centralisation of complex colorectal surgery to specialist surgeons [80], implementation of high-compliance ERAS protocols [41,42,43], and investment in quality improvement programmes all demonstrate significant outcome benefits. Complication rates from AL, SSI, and colovesical fistula are not solely a function of patient physiology; they also reflect the quality of the system within which care is delivered. The centralisation of emergency colorectal surgery to specialist units was associated with significantly lower complication rates and stoma formation compared with non-specialist centres [80]. High-compliance ERAS protocols, including in the emergency setting, have consistently reduced length of stay, complication rates, and costs across multiple studies [41,42,43]. Perioperative beta-blockade, preoperative iron infusion, and nutritional optimisation each represent low-cost, high-impact system-level measures with evidence of meaningful complication reduction [37,39,40]. Taken together, these data highlight that measurable, system-driven improvements in colorectal surgical outcomes are achievable through structured, evidence-based delivery of patient care.

An important consideration in reviewing reductions in complications is also to consider the long-term, functional impact on patients. Complication prevention is not solely an exercise in reducing short-term morbidity and mortality; it is also intended to consider the patient’s quality of life and functional status following colorectal surgery. Anastomotic leak after low anterior resection is amongst the most challenging of complications, not only because of its acute life-threatening potential, but because of its well-established association with the development and severity of low anterior resection syndrome (LARS) [1]. AL disrupts the integrity and neuromusculature of the anastomosis, accelerates fibrosis, and may subsequently require defunctioning or permanent stoma formation, each of which compounds functional morbidity. In this context, strategies have been demonstrated to reduce AL and carry functional significance that extends well beyond the immediate postoperative period [30,36,37,47]. Similarly, the permanent or long-term presence of a stoma carries substantial implications for quality of life, body image, psychological wellbeing, and social functioning.

Stoma reversal rates remain low in practice, and many patients who undergo Hartmann’s procedure in the emergency setting never achieve intestinal continuity [10]. The demonstrated superiority of primary anastomosis over Hartmann’s procedure in selected emergency patients therefore represents not merely a short-term clinical advantage, but a meaningful improvement in long-term functional outcome and stoma-free survival [10,11].

The prevention of SSI also carries long-term functional relevance; deep incisional infections can contribute to prolonged illness, incisional hernia formation, and reduced functional capacity, each of which impairs patients’ return to normal activities and work [4]. Furthermore, perioperative haemorrhage requiring transfusion is independently associated with immunosuppression, impaired wound healing, and increased infective complications, all of which delay functional recovery [37].

The emerging role of prehabilitation through optimising nutritional status, physical fitness and anaemia prior to surgery is key in its association with reduced postoperative morbidity and earlier return to functional independence [45,46]. Remote monitoring technologies and digital health platforms, whilst primarily evaluated as tools for early complication detection, also represent an opportunity to capture and respond to functional decline in the post-discharge period, enabling earlier intervention and rehabilitation [73,74,75,76]. Complication reduction should therefore be understood not merely as an endpoint in itself but as an impactful intervention available to the colorectal surgeon seeking to preserve and optimise the long-term functional wellbeing of their patients.

## 6. Conclusions and Future Directions

Anastomotic leak, surgical site infection, perioperative bleeding and colovesical fistula remain significant sources of morbidity, mortality, and healthcare expenditure following colorectal surgery. The evidence reviewed supports a comprehensive, protocolised approach to their prevention and early diagnosis.

Key preventive priorities include the selective adoption of minimally invasive and robotic surgical approaches; optimisation of patient-level risk factors including obesity, anaemia, nutritional status, and immunosuppression; implementation of ERAS protocols and early mobilisation; utilisation of ICG fluorescence angiography for intraoperative perfusion assessment; prudent use of SEMS bridge-to-surgery in malignant obstruction; and centralisation of care to specialist colorectal surgeons.

Diagnostically, structured surveillance protocols incorporating serial CRP and PCT measurement, drain fluid pH analysis, and early CT imaging, complemented by remote monitoring through wearables and digital health platforms, offer the greatest potential for early AL detection and intervention.

Future research should focus on the prospective validation of digital health diagnostic tools, the role of artificial intelligence in risk prediction and complication surveillance, longer-term oncological outcomes following SEMS bridge-to-surgery strategies, and the potential of novel anastomotic protection devices and adjuncts in high-risk anastomoses. Continued investment in quality improvement initiatives will be key to refine best practice, with the ultimate goal of eliminating preventable complications from colorectal surgery.

## Data Availability

No new data was created or analysed in this study.
